# Synthesis and Characterization of a Novel Nanosized Polyaniline

**DOI:** 10.3390/polym15234565

**Published:** 2023-11-29

**Authors:** Mohd Faizar Banjar, Fatin Najwa Joynal Abedin, Ahmad Noor Syimir Fizal, Norazilawati Muhamad Sarih, Md. Sohrab Hossain, Hakimah Osman, Nor Afifah Khalil, Ahmad Naim Ahmad Yahaya, Muzafar Zulkifli

**Affiliations:** 1 Malaysian Institute of Chemical and Bioengineering Technology, Universiti Kuala Lumpur (UniKL), Alor Gajah 78000, Melaka, Malaysia; faizar.banjar16@s.unikl.edu.my (M.F.B.); fatin.joynal28@s.unikl.edu.my (F.N.J.A.); nafifah.khalil@s.unikl.edu.my (N.A.K.); 2 Centre for Sustainability of Ecosystem & Earth Resources (Pusat ALAM), Universiti Malaysia Pahang, Lebuh Persiaran Tun Khalil Yaakob, Gambang 26300, Pahang, Malaysia; syimir@umpholdings.my; 3 Department of Chemistry, Faculty of Science, Universiti Malaya, Kuala Lumpur 50603, Malaysia; nmsarih@um.edu.my; 4 HICoE-Centre for Biofuel and Biochemical Research, Institute of Self-Sustainable Building, Fundamental and Applied Sciences Department, Universiti Teknologi Petronas (UTP), Seri Iskandar 32610, Perak, Malaysia; sohrab.hossain@utp.edu.my; 5 Faculty of Chemical Engineering & Technology, Universiti Malaysia Perlis, Arau 02600, Perlis, Malaysia; hakimah@unimap.edu.my; 6 Polymer Science Program, Division of Physical Science, Faculty of Science, Prince of Songkla University, Hat-Yai 90110, Songkla, Thailand; 7 Green Chemistry and Sustainability Cluster, Branch Campus, Malaysian Institute of Chemical and Bio-Engineering Technology, Universiti Kuala Lumpur (UniKL), Taboh Naning, Alor Gajah 78000, Melaka, Malaysia; ahmadnaim@unikl.edu.my

**Keywords:** PANI, polymerization, particle size, dispersion, characterization

## Abstract

Polyaniline (PANI) is a conductive polymer easily converted into a conducting state. However, its limited mechanical properties have generated interest in fabricating PANI composites with other polymeric materials. In this study, a PANI–prevulcanized latex composite film was synthesized and fabricated in two phases following chronological steps. The first phase determined the following optimum parameters for synthesizing nanosized PANI, which were as follows: an initial molar ratio of 1, a stirring speed of 600 rpm, a synthesis temperature of 25 °C, purification via filtration, and washing using dopant acid, acetone, and distilled water. The use of a nonionic surfactant, Triton X-100, at 0.1% concentration favored PANI formation in a smaller particle size of approximately 600 nm and good dispersibility over seven days of observation compared to the use of anionic sodium dodecyl sulfate. Ultraviolet–visible spectroscopy (UV-Vis) showed that the PANI synthesized using a surfactant was in the emeraldine base form, as the washing process tends to decrease the doping level in the PANI backbone. Our scanning electron microscopy analysis showed that the optimized synthesis parameters produced colloidal PANI with an average particle size of 695 nm. This higher aspect ratio explained the higher conductivity of nanosized PANI compared to micron-sized PANI. Following the chronological steps to determine the optimal parameters produced a nanosized PANI powder. The nanosized PANI had higher conductivity than the micron-sized PANI because of its higher aspect ratio. When PANI is synthesized in smaller particle sizes, it has higher conductivity. Atomic force microscopy analysis showed that the current flow is higher across a 5 µm^2^ scanned area of nanosized PANI because it has a larger surface area. Thus, more sites for the current to flow through were present on the nanosized PANI particles.

## 1. Introduction

Conductive polymers (CPs) are organic materials with unique electrical and optical properties. They can be made using simple, affordable methods and assembled into structures with multiple functions [1]. The five main types of CPs are polyacetylene, polythiophene, polypyrrole, polyphenylene, and polyaniline (PANI) [2]. The conductive properties of these CPs derive from the conjugated π system of the polymer backbone structure [3]. The selection of PANI offers good conductivity, antimicrobial properties, corrosion inhibition, and magnetic properties [4,5]. The synthesis of PANI-based composites has recently attracted a lot of interest among researchers because of the possible new properties that could be achieved through combining PANI with other materials such as templates, hosts, and substrates [6]. PANI–cellulose composites are potential antimicrobial textile materials in the biomedical field [3].

Synthesizing PANI with metal oxides has been performed by researchers seeking humidity sensors, biomedicine, and antioxidants [7,8]. Several plastics have been used to fabricate conductive composites with elasticity properties using PANI. These composites have several applications, including their potential for use as chemical sensors, solar cells, capacitors, and stretchable electronic devices [9]. PANI can be mixed with other polymeric materials such as elastomers to overcome its lack of mechanical properties [10,11,12,13]. Prevulcanized latex (PVL) is a good choice for fabricating latex composite materials because it has good mechanical properties and can be stored for prolonged periods at ambient temperature [14].

The synthesis of fillers in the nanodimensions allows for the optimization of intrinsic properties, as a larger aspect ratio is expected. The mechanism of PANI synthesis requires the identification of the affecting parameters because different properties are obtained by varying the parameter settings. The main objective of this work was to produce PANI in nanodimensions or smaller than 1 micron. PANI is synthesized via chemical oxidative polymerization with the addition of surfactants. Chemical oxidative polymerization is the easiest approach to use for synthesizing PANI [11,15,16,17]. Most papers yield micron-sized (µm) PANI synthesized via the conventional chemical oxidative polymerization method, which is unsuitable for advanced nanotechnology applications [18,19,20,21]. The oxidizing agent used to polymerize aniline was ammonium persulfate (APS), and surfactants such as anionic sodium dodecyl sulfate (SDS) and nonionic Triton X-100 (TX100) were used to enhance the dispersibility of PANI, in addition to controlling the particle size of PANI. The colloidal and dispersion stability of PANI dispersions is a valuable detail for tracking the quality based on homogeneity and thus producing a stable dispersion. Mixing this nanoparticle with a host material such as latex has a broad range of beneficial applications, such as hybrid antibacterial and conductive coatings for textiles [22,23]. The use of nanoparticle-based technologies provides opportunities for improving efficiency and sustainability.

The PANI synthesis parameters affect the morphology and particle size of nanosized PANI [24,25,26,27]. Reviews regarding PANI particle size mostly cover scanning electron microscopy (SEM) analysis without considering measurements via the dynamic light scattering (DLS) method [28,29]. The parameters of PANI polymerization discussed herein include the aniline to APS initial molar ratio (*r*), stirring speed of synthesis, synthesis temperature, the PANI purification method, the PANI washing method, and the use of different surfactants. PANI polymerized with secondary growth is irregularly shaped in the absence of surfactants as soft templates, as well as morphological and particle size control [30,31,32]. Surfactants act as templates in polymer synthesis, and their polarity influences the particle size of the resulting polymers. Jamdegni et al. revealed that using anionic SDS and nonionic TX100 affects polymer size and shape [18]. A higher stability of the dispersion of synthesized nickel nanoparticles was obtained in the presence of anionic SDS compared with the use of a cationic surfactant [33].

PANI powder was synthesized in this study using predefined parameters. The parameters monitored included *r*, stirring speed, temperature, purification method, washing method, and surfactant effect. Optimized parameters were determined in stages according to the results of our Fourier transform infrared spectroscopy (FTIR) analysis of PANI quality, the smallest particle size of PANI with a range lower than 1 µm, and a good dispersion of colloids, as no phase separation was observed. The outcome of this phase provides the optimum parameters for synthesizing PANI powder.

PANI synthesized into the nanodimensions is beneficial when the surface-to-volume ratio is high. The four most explored methods for fabricating PANI are the use of a hard template, the use of a soft template, electropolymerization, and electrospinning [18]. Among these options, the route of chemical synthesis using a soft template is preferable, and this is the predominant approach in many studies because of its low cost and ability to facilitate the easy recovery of PANI [30,32,34]. The soft template method eases the purification of PANI and is a cheaper and more efficient technique [31].

## 2. Methodology

### 2.1. Materials

Nanosized PANI was synthesized via chemical oxidative polymerization with and without a surfactant. The list of chemicals used is summarized in Table 1. Preparation was performed under acidic conditions (0.2 M hydrochloric acid, HCl) with APS as the oxidizing agent. Two types of surfactants were used: anionic SDS and nonionic Triton X-100 (TX100). For sample washing, 0.2 M HCl and acetone were added. PVL was used as the host material.

### 2.2. Synthesis of Nanosized PANI

**Figure 1:**
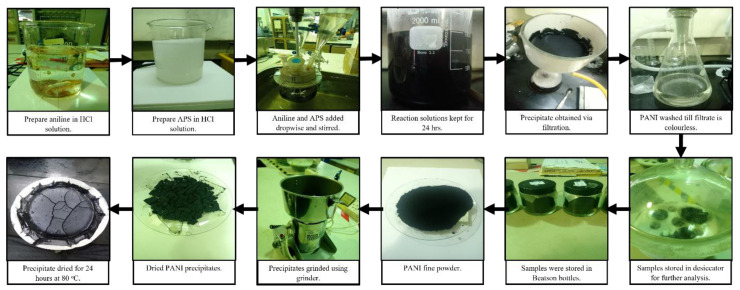
Schematic of PANI synthesis.

Nanosized PANI was synthesized via chemical oxidative polymerization in a controlled temperature reactor. The reactants were added dropwise and simultaneously using a separation funnel. A schematic of the PANI synthesis process is shown in Figure 1. 

### 2.3. Phase 1: Parameters of PANI Synthesis

To synthesize PANI in nanosized form, several parameters, all of which will be discussed in more detail later in this section, must be considered. Standardized methods for synthesizing PANI nanoparticles are limited, as the optimal approaches vary depending on the intended application of PANI. Moreover, the control of PANI particle size is least defined in methods not well suited for producing nanoparticles in the colloidal size range. The precursor method used in this study was adapted from the International Union of Pure and Applied Chemistry (IUPAC) technical report by J. Stejskal in 2002, in which PANI was prepared using a standardized approach involving many institutions [35]. The reference sample had the parameter conditions listed in Table 2.

#### 2.3.1. Initial Molar Ratio of Aniline/APS (*r*)

The first parameter that must be considered is the ratio of the reactants aniline monomer and APS as the polymerization initiator or oxidizing agent. Values of *r* of 1 (0.2 M:0.2 M) and higher (0.3 M:0.1 M) were chosen for synthesizing PANI. The produced PANI precipitate was filtered, washed repeatedly, dried, and ground to obtain the final powdered product. The formulations were investigated based on the yield of PANI and FTIR spectra.

#### 2.3.2. Stirring Speed

The effect of stirring speeds or agitation intensities during synthesis on the particle size of PANI was examined. Two speeds were chosen: 600 and 1200 rpm.

#### 2.3.3. Synthesis Temperature

The synthesis temperature also affects the properties of PANI. Synthesis at a low temperature (0 °C) is the most common approach, as used in a few studies in the literature. Numerous other sources apply synthesis at ambient temperature or 25 °C [36,37]. Effective temperature control was achieved using minimal exposure to the open area and an insulated container at 0 °C, while the temperature of the water bath was set at 25 °C. The effect on particle size was studied and will be discussed later in this paper.

#### 2.3.4. Purification Method

PANI was purified using two approaches: vacuum filtration and heating. Our comparison of the methods was based on the convenience they offered in terms of product loss, time, and effort. Vacuum filtration uses a Buchner funnel and pump to promote vacuum conditions, forcing filtrate separation from PANI solution at room temperature. The heating method separates the solution obtained from PANI synthesis via evaporation at 80 °C. The optimal method was determined from the outcome of PANI FTIR and particle size.

#### 2.3.5. Washing Method

During the filtration process, PANI was collected on filter paper and washed, while the filtrate was collected in a flask. The substances used as the washing agent included 0.2 M HCl, 0.2 M methanol, and 0.2 M acetone at three consecutive cycles. Excess distilled water was used for final washing until the filtrate was colorless. The order of the washing substances was as follows: 0.2 M HCl followed by 0.2 M acetone and distilled water. Acetone was replaced with 0.2 M methanol to investigate the importance of PANI washing. The effectiveness of each substance in washing was monitored according to the FTIR analysis of the filtrate. The particle size of PANI at each washing stage was further monitored to investigate the effect of washing on particle size.

#### 2.3.6. Surfactant in Synthesis

A surfactant (SDS or TX-100) was added to the reactor at a concentration of 0.1% *w*/*w*. The solutions were then stirred for 45 min to disperse the surfactant. Aniline and APS were then added dropwise into the surfactant solution until the reaction mixture changed from milky white to dark green. After the reactant was consumed, the reaction solution was stirred for 24 h to complete polymerization. The solution was then transferred to a beaker, sealed with aluminum foil, and left for another day. The process proceeded by filtering and washing with the same approach, with storing and drying being carried out accordingly. The sample was ground into powder form and stored in a Beatson bottle for further analysis. The procedure was repeated using different concentrations of the surfactant: 0.5% *w*/*w*, 1.0% *w*/*w*, and 1.5% *w*/*w*.

### 2.4. Characterization of PANI Powders and Dispersion

#### 2.4.1. FTIR Analysis of PANI

After drying in an oven for 2 h at 60 °C to remove any remaining moisture, the PANI powder was subjected to FTIR analysis to determine the functional groups present. A Nicolet iS10 FTIR spectrometer with attenuated total reflection was used to confirm the presence of the key characteristics of the substance. The spectral range was from 400 to 4000 cm^−1^ with 2 cm^−1^ resolution under ambient conditions. The acquired data were recorded using ASTM E168 [38] in the absorbance mode. Liquid samples such as the PANI washing filtrate, 0.2 M acetone, and HCl were analyzed using a Perkin Elmer FTIR instrument (Waltham, MA, USA).

#### 2.4.2. Particle Size Analysis of PANI

The particle size of the PANI powder was analyzed using a Zetasizer 2590 (Worcestershure, UK). The prepared sample had a concentration of 0.012% in distilled water and was inserted into a specified cuvette at an adequate concentration of approximately 1 mL. Measurements were taken in triplicate immediately before the particles underwent sedimentation. Sonication of the samples was performed for 1 min at 30 °C.

#### 2.4.3. UV-Vis Absorption Spectra of PANI

The PANI powder was dispersed in N-methylpyrrolidone (NMP) at a fixed PANI concentration of 0.4%. The UV-Vis spectra of the PANI solutions in NMP were recorded from 250 to 900 nm using a Shimadzu UV-1900i spectrophotometer (Kyoto, Japan).

#### 2.4.4. Morphology of PANI

The morphology and structure of the PANI powder were identified using SEM analysis. Samples of PANI powder in micron size and nanosize were selected as measured from the DLS analysis. The samples were dried beforehand using a cold vacuum to remove moisture from the powder. The samples were coated with a thin layer of gold before images were taken using an accelerating voltage of 10 kV. SEM images were taken at a scale of 10 µm × 10 µm. The size of PANI powder was measured using ImageJ v4.3.4.Build22 imaging software.

#### 2.4.5. Conductivity of PANI

The conductivity of the SEM-analyzed PANI powder was measured using an NX10 atomic force microscope in the conductive mode. Measurements were taken at multiple areas over a scanning area of 5 µm × 5 µm. Atomic force microscopy (AFM), in the conductive mode, was performed by placing the PANI powder on a standard metal disk, and attachments were made using conductive epoxy glue to ensure that the sample remained in the scanning area and the complete flow of the current. The voltage was 1.0 V, and a standard solid C-AFM probe was used as the cantilever.

## 3. Results and Discussion

### 3.1. Synthesis of PANI

PANI was synthesized via chemical oxidative polymerization because of its convenience in preparation and processing, as addressed by many studies [2,15,16,39]. PANI was polymerized using aniline as the monomer, APS as the oxidizing agent, and HCl solution to produce high acidity media.

### 3.2. Determination of r

*r* is a vital parameter in PANI polymerization [15,39]. Although *r* can be determined directly from the stoichiometric equation of the PANI polymerization reaction, adjustment to other conditions is often considered to obtain properties and further clarify the significance of *r*. The concentration of HCl as a reaction medium was kept at 0.2 M, with a synthesis temperature of 0 °C–5 °C in PANI polymerization, as recommended in numerous research papers [40,41]. The stirring speed was 800 rpm throughout the synthesis period. A comparison was made between equal and unequal molar ratios, where 0.2 M aniline and 0.2 M APS were used to obtain *r* = 1, and 0.3 M aniline and 0.1 M APS were used to obtain *r* = 3. The higher molar concentration of aniline was chosen as a comparison target because of the consensus in the literature that advantageous properties are attainable, such as optimized fiber morphology [27]. The produced PANI precipitates were filtered, washed repeatedly, dried, and ground to obtain the final powdered product. The formulations were investigated based on the yield of PANI and FTIR spectra.

#### 3.2.1. Yield of PANI

Table 3 presents the reaction yield for PANI, which is 90.39% for *r* = 1 and 34.72% for *r* = 3. This drop was expected because a higher amount of aniline remains in the reaction media, whereas APS has been consumed, forming a limited amount of PANI [39]. Equimolar formulations have higher yields than unequal molar formulations, suggesting that both reactants were depleted simultaneously, while termination occurred for higher *r*, as the oxidant was used up [40]. The slight reduction in yield from the equimolar formulation was possibly due to losses mainly during the purification of PANI, where a repeated cycle of washing and filtering was applied. This result shows that *r* has a pronounced effect on the yield of PANI and that the mode of synthesis is preferable at *r* = 1.

#### 3.2.2. FTIR of PANI with Equal and Unequal Molar Ratios of Aniline/APS

**Figure 2:**
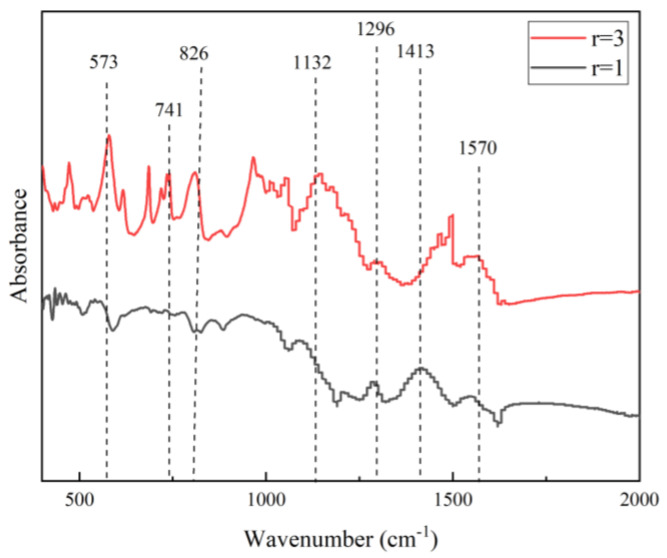
FTIR of PANI synthesized with different *r* values.

The FTIR spectra of PANI synthesized with *r* = 1 and higher are shown in Figure 2. The two peaks at 1413 cm^−1^ and 1570 cm^−1^ are ascribed to the C=C stretching modes of the benzenoid and quinoid rings in the PANI chain, respectively [18]. The PANI characteristic peaks observed at 1132 cm^−1^ and 1296 cm^−1^ reflect a charged amine structure between the aromatic ring and the C–N stretching vibrations of aromatic amines, respectively [42]. The peak at 826 cm^−1^ corresponds to the 1,4-disubstituted quinoid rings. The strong peak at 573 cm^−1^ corresponds to the sulfate counter ion of SDS present at *r* = 3. The peak at 741 cm^−1^ corresponds to the wag vibration of the aniline monomer N–H group [43]. In this formulation, PANI was formed in limited amounts relative to the limiting amount of APS, leaving the aniline monomer unused.

Although multiple washings were applied, these unreacted species remained in the final product [18]. The absence of unfavorable constituents was a key factor in the high-quality PANI synthesis achieved using the *r* = 1 formulation. This distinction in the properties of PANI due to the alteration in *r* results in the PANI gaining different properties, as highlighted in the FTIR analysis and the yield of PANI.

### 3.3. Stirring Speed of PANI Synthesis

The effect of stirring speed on the quality of PANI and the particle size was monitored at speeds of 600 and 1200 rpm. The agitation of reactants during the reaction is important for the uniform distribution of substances and a reduced possibility of agglomeration. An equimolar ratio of aniline to APS was maintained to determine the optimum stirring speed. The quality of the PANI was assured using FTIR and particle size distribution data.

#### 3.3.1. Effect of Stirring Speed on the FTIR

**Figure 3:**
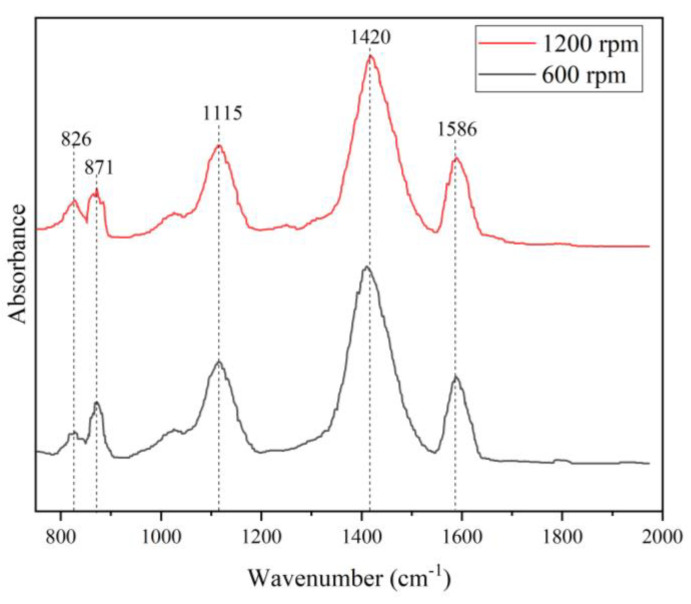
FTIR of PANI synthesized at different stirring speeds.

The FTIR spectra of the PANI synthesized at stirring speeds of 600 and 1200 rpm are shown in Figure 3. The double peaks at 1586 cm^−1^ and 1420 cm^−1^ correspond to the C=C stretching modes of the benzenoid and quinoid rings, respectively. The peak at 1115 cm^−1^ indicates the presence of an amine structure between the aromatic rings, while 871 cm^−1^ and 826 cm^−1^ correspond to the 1,4-disubstituted quinoid ring. The obvious assignment peaks of PANI were present for both stirring speeds and showed almost identical trends. The quality of PANI was not substantially affected by changing the stirring speed, which only involves mechanical action during the polymerization of PANI. A similar FTIR trend was reported by Kamarudin in 2021, where PANI synthesized with different stirring speeds showed no obvious differences [44].

#### 3.3.2. Effect of Stirring Speed on Particle Size

**Figure 4:**
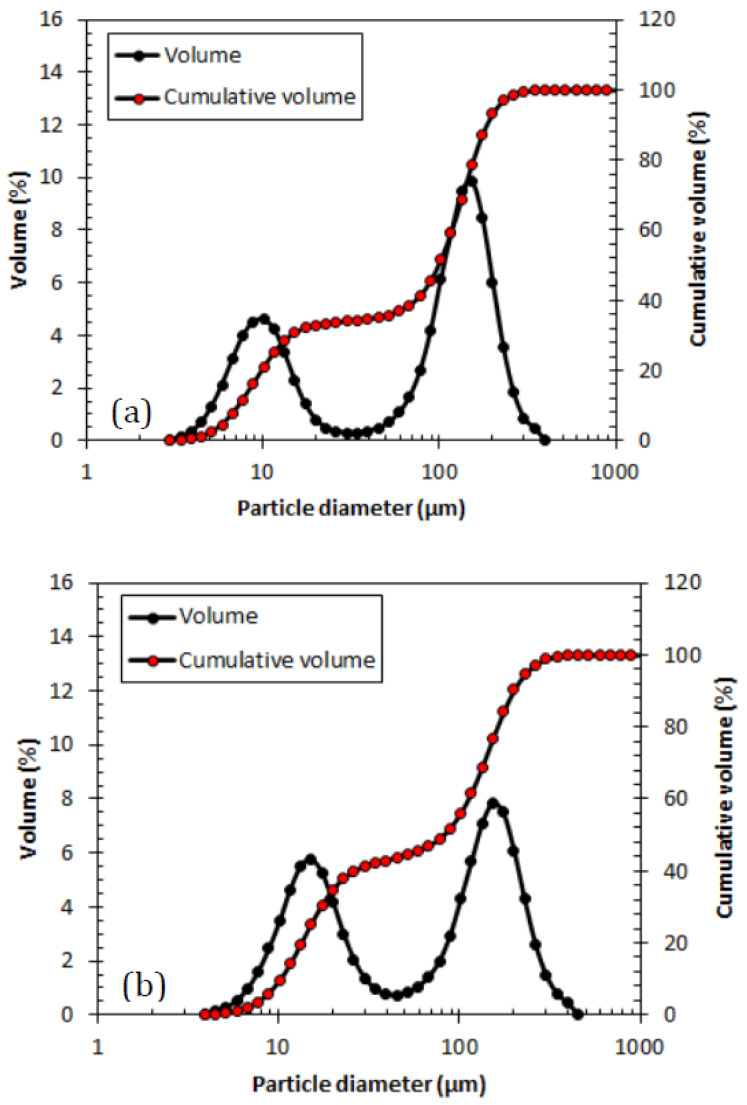
Particle size distribution of PANI synthesized at (**a**) 600 rpm and (**b**) 1200 rpm.

Stirring speed had an unsubstantial effect on the FTIR results. A similar conclusion was reached using the supporting data of particle size analysis for both conditions, as displayed in Figure 4, which shows the plots of volume and cumulative volume versus particle diameter. Both samples show a binomial distribution. At a stirring speed of 600 rpm, 65% of the particle population is large, whereas at 1200 rpm, the populations of large and small particles are equal.

No substantial difference in particle size was observed when the stirring speed was altered during PANI synthesis. The particle diameter shows an almost identical particle size for smaller and larger particle populations, as shown in Table 4. The selection of a lower stirring speed is preferable to reduce energy consumption and the cost of utilities in synthesizing PANI.

### 3.4. Synthesis of PANI at Different Temperatures

The synthesis of PANI was performed at 0 °C and 25 °C. Surfactants were omitted from the synthesis process due to the effect of the Krafft temperature factor on the performance of surfactant function ability. Micelle formation occurs above the Krafft temperature of SDS, which is 25 °C and above [32]. This alteration was made in the synthesis of PANI without surfactants to elucidate the effect of particle size. FTIR analysis was not performed because the fingerprints of PANI synthesized at different temperatures were identical [31].

#### Effect of Synthesis Temperature on Particle Size

**Figure 5:**
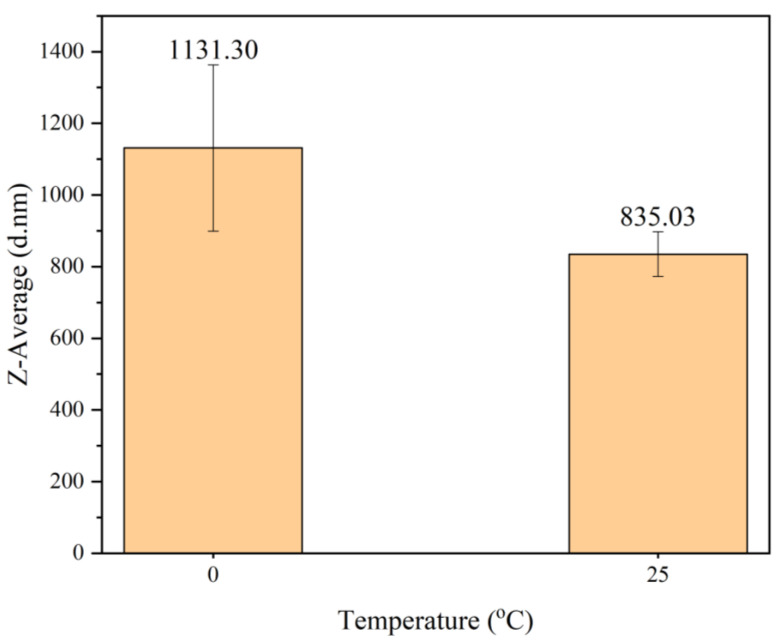
Particle size of PANI synthesized at different temperatures.

The results of a particle size analysis of PANI synthesized at 0 °C and 25 °C are shown in Figure 5. The particle sizes of PANI synthesized at 0 °C and 25 °C were 1131.30 nm and 835.03 nm, respectively. According to Aribowo in 2018, synthesizing PANI at lower temperatures increased the molecular weight and crystallinity [31]. Because the polymerization of PANI is exothermic, the reaction period is bound to be longer at lower temperatures and vice versa [45]. Holding a lower temperature results in the formation of large particles because an increase in crystallinity increases crystallite size; hence, an increase in particle size was expected [31,32]. Because it resulted in a smaller particle size, faster reaction time, suitability for implementing SDS in polymerization, and better temperature control, PANI was further synthesized at 25 °C instead of 0 °C. These advantages are important, particularly for the large-scale production of PANI.

### 3.5. Purification Method for PANI

PANI synthesized after 24 h produces a solution containing PANI, unreacted reactant, oligomers, and mostly a solution of acidic HCl media. The purification of PANI was performed via filtration or heating. The quality of the purified PANI was determined using FTIR and particle size.

#### 3.5.1. Effect of PANI Purification Method on the FTIR

**Figure 6:**
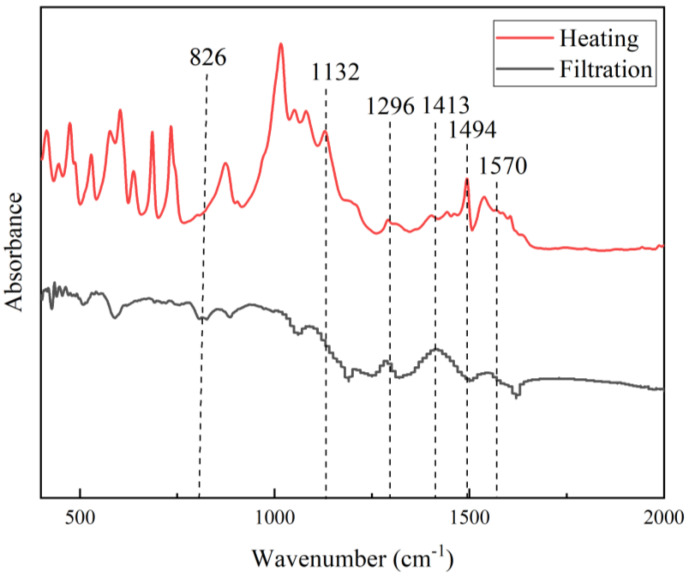
FTIR spectra of PANI purified via heating or filtration.

The FTIR spectra of PANI purified via filtration or heating are shown in Figure 6. Both spectra exhibit the characteristic peaks of PANI, but they differ notably in some shifting and absorbance intensities. The peak at 1494 cm^−1^ showed an increased intensity, indicating a higher quinoid structure upon heating [42]. The peak at 1413 cm^−1^ is ascribed to the benzenoid ring stretching mode of C=C. A higher intensity was also observed at 1296 cm^−1^, while a lower intensity was observed at 806 cm^−1^. Purifying PANI via heating results in many additional unidentified peaks. Other substances were produced because of the prolonged heating of PANI. Heating has a much lower tendency for product loss, resulting in a better yield of PANI.

#### 3.5.2. Effect of Purification Method on Particle Size

**Figure 7:**
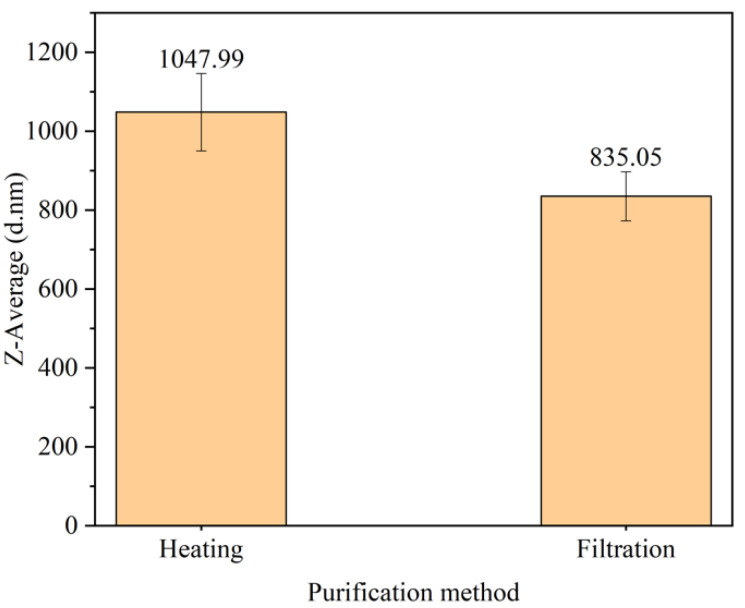
Particle size analysis of PANI using different purification methods.

The particle sizes of PANI purified via heating and filtration are displayed in Figure 7. Purifying PANI via heating produces a larger particle size of 1047.99 ± 98 nm compared to 835.05 ± 62.12 nm using the filtration method. In Figure 6, the additional peaks in the FTIR data when the heating method was used are attributed to the presence of additional substances in PANI. This result explains the presence of other unknown substances that contribute to the size of PANI. Differences in the yield were also observed, with heating providing a higher yield than the filtration method, as displayed in Table 5. During heating, product loss was minimized because the process occurred in the same batch [34]. The filtration method was preferred because of the presence of characteristic PANI peaks and smaller particle sizes.

### 3.6. Washing Method for PANI

PANI was washed using different washing substances, such as 0.2 M HCl, 0.2 M acetone, 0.2 M methanol, and distilled water. The filtrates were analyzed via FTIR to confirm the presence of impurities and the washing efficiency. The particle size of PANI was also monitored and analyzed accordingly to investigate the effect of washing on particle size.

#### 3.6.1. Effect of PANI Washing Method on the FTIR

**Figure 8:**
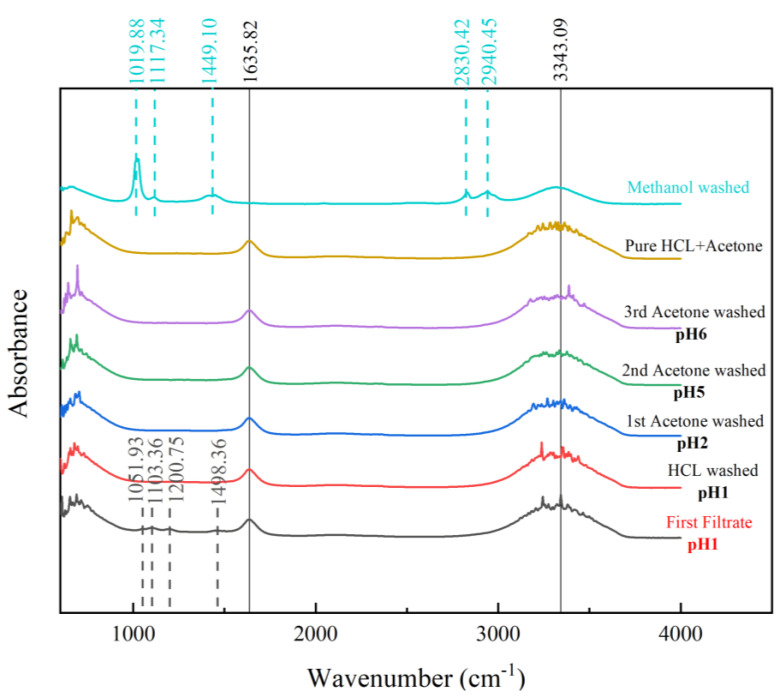
FTIR spectra of PANI filtrates.

The selection of the washing method for PANI is important for removing unreacted substances, intermediates, and by-products [31]. The removal of these undesired substances is necessary to produce good-quality PANI with beneficial properties. The FTIR of the filtrate was investigated at each stage of washing with specified arrangements, as displayed in Figure 8.

The first filtrate was defined as the filtrate from the PANI reaction solution. The important peaks of the undesired substances are highlighted by the blue dashed lines. This solution contains unreacted benzenoid, as the benzenoid ring stretching peak at 1498.36 cm^−1^ is in the spectrum. The peak at 1200.75 cm^−1^ indicates a 1,4-ring and 1,3-disubstituted and monosubstituted benzene. Because of oligomers or intermediate products of PANI polymerization, the peak of a phenazine-type ring was identified at 1103.36 cm^−1^. This species must be removed, as its conductivity was lower, on the order 10^−7^ to 10^−9^, and it could lower the conductivity of PANI [46]. Counterions such as hydrogen sulfate (HSO_4_^−^), present because of the use of APS in the synthesis process, produced a peak at 1051.93 cm^−1^.

The washing process continued using 0.2 M HCl to create uniform doping across the PANI chain and remove residual monomers, soluble oligomers, and by-products such as HSO_4_^−^ [47]. According to the spectra of the HCl-washed PANI, these species were removed successfully, as the respective peaks vanished. Similar outcomes were obtained after washing with acetone was applied three times. Acetone itself is needed to overcome the further aggregation of PANI during the drying process to prevent the further polymerization of PANI [44]. The FTIR of the HCl and acetone mixture was compared to confirm that the filtrate was clean and free from any traces of other substances. The third cycle of washing with acetone removed all traces of HSO_4_^−^, 1,3-disubstituted benzene, and oligomers from the PANI sample, as evidenced by the identical spectral trends of the final filtrate and the pure HCl–acetone mixture. This result indicates that the PANI sample was effectively washed.

The extra peaks identified at 1019.88 cm^−1^, 1117.34 cm^−1^, 1449.10 cm^−1^, 2830.42 cm^−1^, and 2940.45 cm^−1^ were mostly due to secondary amine salts. Thus, it is an unfavorable washing agent, as some studies have suggested [30,42]. The presence of amine salts was suspected to result from PANI chain deterioration because the PANI structure comprises repeating units of quinoid rings and benzenoid rings connected by imines and amines [29]. Using methanol is unfavorable for washing the PANI produced in this study.

#### 3.6.2. Effect of PANI Washing Method on Particle Size

**Figure 9:**
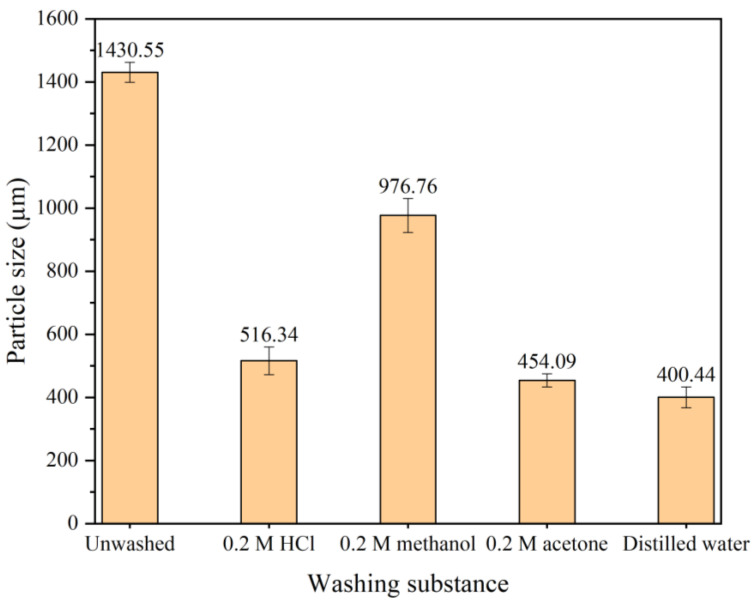
Particle size analysis of PANI washed using different substances.

The particle sizes of PANI washed with different substances are displayed in Figure 9. The results show that the particle size of unwashed PANI is 1430 µm. As the washing process proceeds using other substances, the particle size decreases to 400 µm at the final washing with excess distilled water. The larger size of unwashed PANI was attributed to the accumulation of oligomers and unreacted aniline in the size evaluation using DLS. Washing with 0.2 M methanol reduced the PANI particle size to 976 µm, less efficient than 0.2 M acetone. The use of acetone helps prevent PANI aggregation during drying by removing the oligomers present during the PANI polymerization process [47]. This result explains the substantial particle size reduction from unwashed PANI to the use of methanol. According to the FTIR results and particle size analysis, a washing approach using 0.2 M HCl followed by 0.2 M acetone and excess distilled water is preferable.

### 3.7. Effect of Different Surfactants in the Synthesis of PANI

PANI was synthesized with a surfactant at different loadings to alter PANI particle size. Two surfactants were used (anionic SDS and nonionic TX100) at loading values of 0.1%, 0.5%, 1.0%, and 1.5%. The effect of the surfactants on PANI synthesis was elucidated using FTIR, particle size analysis, and the observation of the dispersions in distilled water for seven days.

#### 3.7.1. Effect of Different Surfactants in Synthesis on the FTIR Spectra

**Figure 10:**
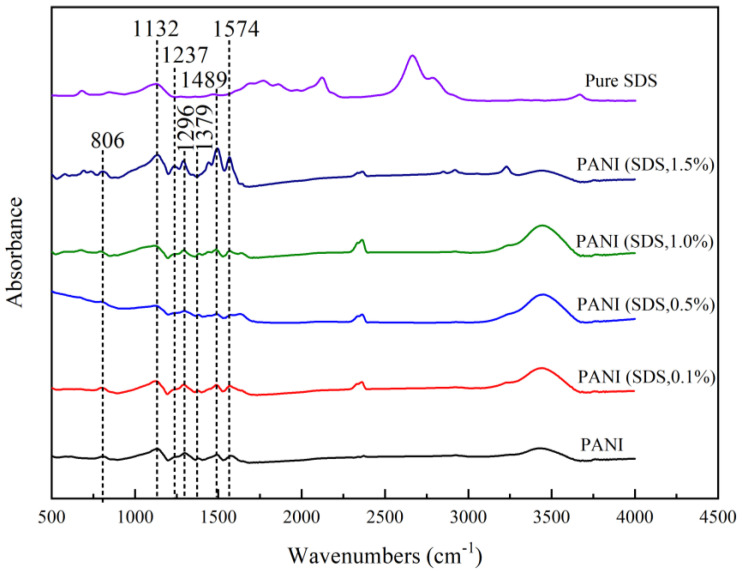
FTIR spectra of PANI–SDS.

The representative spectra of the PANI powder samples synthesized with different concentrations of SDS are illustrated in Figure 10. The presence of the broad vibration band of water molecules at 3425.65 cm^−1^ in all spectra indicates high humidity during measurements [32]. The bands at 1574 cm^−1^ and 1489 cm^−1^ refer to quinoid (Q) and benzenoid (B) ring stretching vibrations, respectively [42]. A small band attributed to the stretching vibration of the C–N group near the quinoid ring can be observed at 1379 cm^−1^. The band that can be observed at 1296 cm^−1^ is due to C–N 1 washing vibrations of aromatic amines linked with para-link aniline units, a common and well-established PANI chain position linkage. The peak at 1237 cm^−1^ is attributed to C–N vibration in the strong band, representing a vibrational mode of the charged imine structure (-NH+=) between quinoid and benzenoid rings. This peak indicates the presence of a positive charge in the backbone of PANI or in the conducting emeraldine form. This peak also exists in the spectrum of the SDS powder sample, ascribed as a -CH in-plane vibration [18]. The band at 806 cm^−1^ observed in the spectrum of the PANI sample synthesized with and without PANI is attributed to the 1,4-disubstituted ring or quinoid ring deformation. The spectra of PANI and PANI synthesized via SDS show almost identical trends, denoting that the surfactant was removed in the final product. Washing PANI with 0.2 M HCl, acetone, and distilled water is important in surfactant removal, as no surfactant fingerprint was imparted in the FTIR. Similar observations were also made when using TX100 as a surfactant in PANI synthesis.

**Figure 11:**
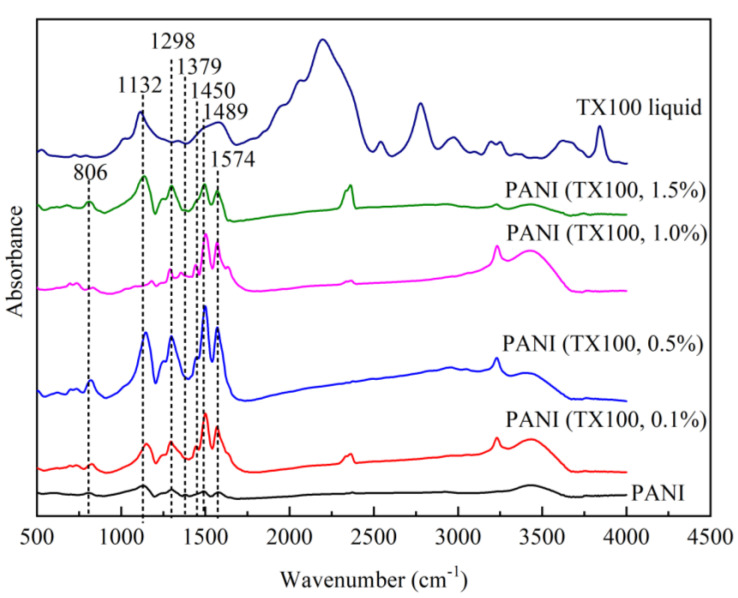
FTIR of PANI–TX100.

The FTIR spectra of liquid Triton TX100 and PANI synthesized with and without the presence of a nonionic surfactant are shown in Figure 11. The PANI produced in the presence of a surfactant and the PANI formed in self-assembly or without a soft template have the same characteristic peaks. The strong band at 1498 and 1574 cm^−1^ corresponds to the stretching of the benzenoid and quinoid rings, respectively. The small band at 1450 cm^−1^ is attributed to the skeletal C=C stretching vibration of the aromatic ring [18]. Another weak band at 1379 cm^−1^ indicates the stretching vibration of the C–N group adjacent to the quinoid ring, also expected for the PANI IR bands of aromatic amines with para-linked aniline monomer units [48]. The strong band at 1132 cm^−1^ represents the vibrational mode of a positively charged amine structure between a benzenoid–benzenoid ring and benzenoid–quinoid ring [49]. Its presence in the TX100 liquid spectra suggests the -CH in-plane vibration [34]. The band at 806 cm^−1^ is due to quinoid ring deformation or the 1,4-disubstituted ring, confirming para-coupling in the PANI chains [26].

#### 3.7.2. Effect of Different Surfactants in Synthesis on the UV-Vis Spectra

**Figure 12:**
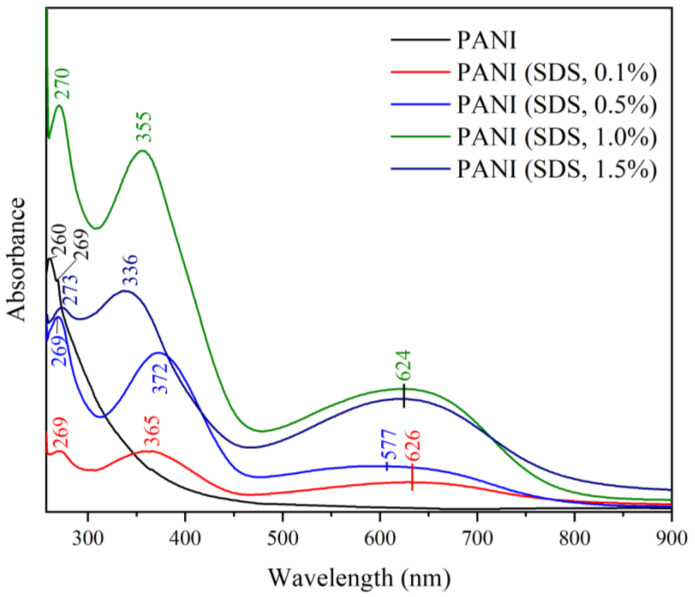
UV-Vis of PANI–SDS.

**Figure 13:**
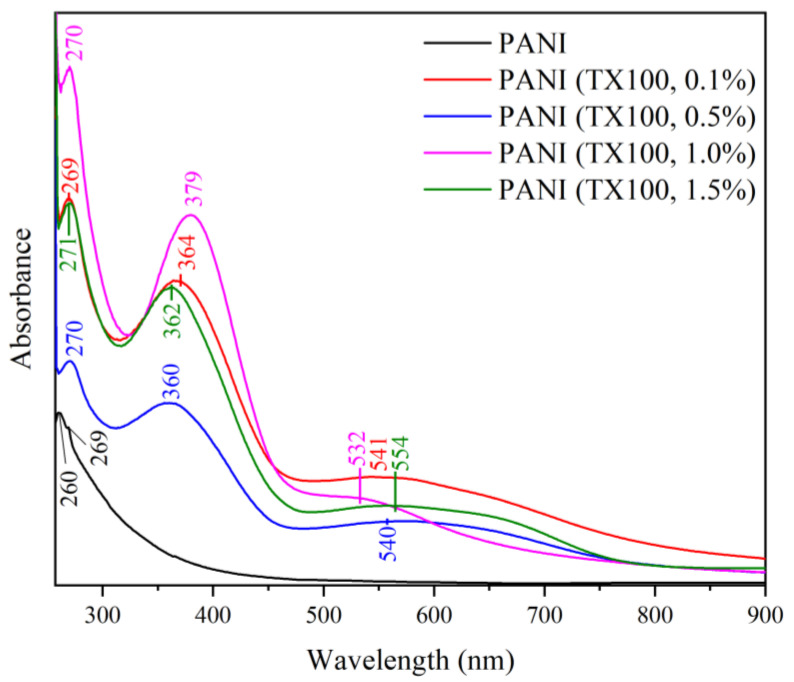
UV-Vis of PANI–TX100.

The UV-Vis spectra of PANI synthesized with different loadings of a surfactant, namely, SDS and TX100, are shown in Figure 12 and Figure 13, respectively. Two absorption bands at 260 and 269 nm can be observed in the UV-Vis spectrum of PANI synthesized freely without a template or surfactants. These bands are attributed to the π–π* transitions of the benzenoid ring of the PANI backbone [45,50,51]. The washing process increased the solution pH and decreased the acidity of the PANI powder. This decrease in acidity reduced the doping of the PANI backbone. This reduction is corroborated by the absence of an absorption peak in the range of 400 to 450 nm, indicating the doping level of PANI, which is typical in PANI salt [52]. PANI synthesized with SDS and TX100 displays a typical PANI emeraldine base spectrum comprising two peak ranges from 330 to 380 and 510 nm to 610 nm. The first peak range is assigned to a π–π* transition of the phenyl ring, which corresponds to the excitation of the amine nitrogen (-NH-) near the benzenoid ring of PANI. The second peak range is ascribed to an n–π* transition corresponding to the excitation of the imine nitrogen (-N=) of the quinoid segments [53]. This trend is consistent with reports of PANI dispersion in NMP in multiple studies in the literature [50,51].

#### 3.7.3. Effect of Different Surfactants in Synthesis on Particle Size

**Figure 14:**
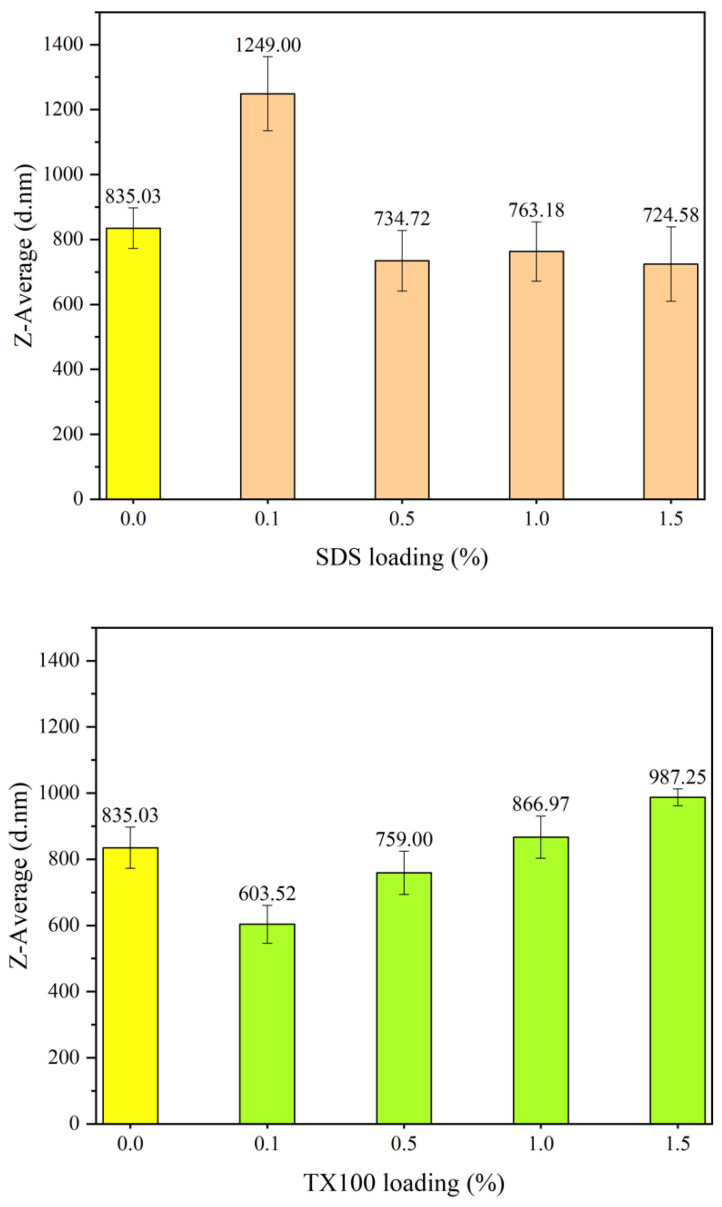
Particle size analysis of PANI synthesized using SDS and TX100.

The top of Figure 14 presents the particle sizes of the respective PANI synthesized via the application of the anionic surfactant SDS. The bar in yellow colour indicated for PANI synthesized without surfactant while brown colour and green colour meant for SDS and TX100, respectively. The particle diameter (measured in nm) indicates the Z-average or the mean of the particle size distribution. The control sample, PANI synthesized without the presence of a surfactant, shows a particle size of 835.03 nm. The average particle size of PANI was considerably increased to 1.25 μm when 0.1% SDS was used. This result exceeds the size limit of a colloidal particle, which must be lower than 1.00 µm. Further increasing SDS loading in synthesis slightly reduced the particle size, with the lowest being recorded at 1.5% loading. SDS acts as a surfactant to assist in reducing PANI particle size at certain loadings [30,31,32]. Consequently, the SDS surfactant was less effective in reducing PANI particle size, and even colloidal PANI particles were produced at higher surfactant loadings.

This result contrasted with the performance of nonionic Triton TX-100, which substantially reduced the PANI particle size to as low as 603.52 nm, as displayed in Figure 14. TX100, as a nonionic surfactant, reduces the particle size of PANI by producing steric repulsion between particles, which prevents agglomeration [48]. When the loading of TX100 is above 1.0%, the particle size increases to a larger size than the control sample. The particle size of PANI was substantially reduced by Triton TX100 at low concentrations, with optimum results at a concentration of 0.1%. This condition is favorable for synthesizing nanosized PANI.

**Figure 15:**
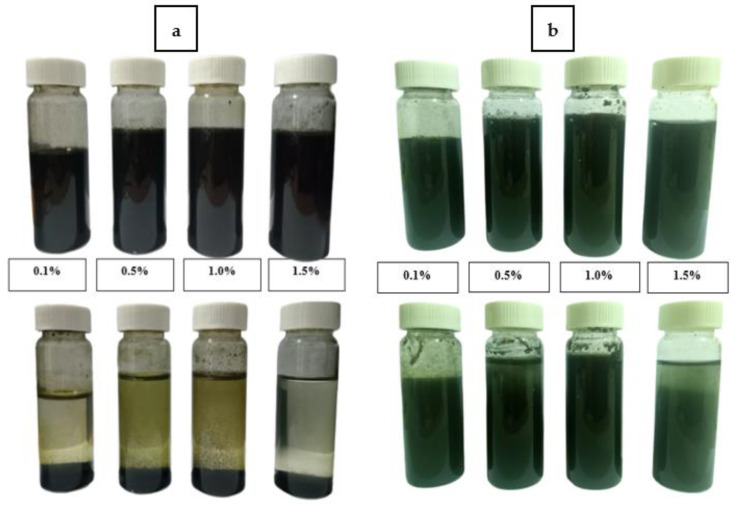
Dispersion of (**a**) PANI–SDS and (**b**) PANI–TX100 in distilled water at pH 7 on day 1 (above) and day 7 (below).

Sample homogeneity was clarified by dispersing a small amount of PANI powder in distilled water and leaving it for seven days to observe the stability of the dispersion upon settling, as shown in Figure 15a,b for PANI–SDS and PANI–TX100, respectively. PANI–SDS dispersion produces foam at the surface, indicating a small amount of SDS surfactant. After being left inside vials for seven days, 0.1%, 1.0%, and 1.5% PANI–SDS dispersions underwent sedimentation, indicating large particles of more than 1.0 µm. An unstable dispersion and poor dispersibility should result from a larger particle size, as observed via DLS. The particles settled due to gravity over time. The PANI–TX100 dispersions were more stable, as no phase separation was observed. This result was supported by the smaller particle size of PANI.

### 3.8. Morphologies of PANI Powders

**Figure 16:**
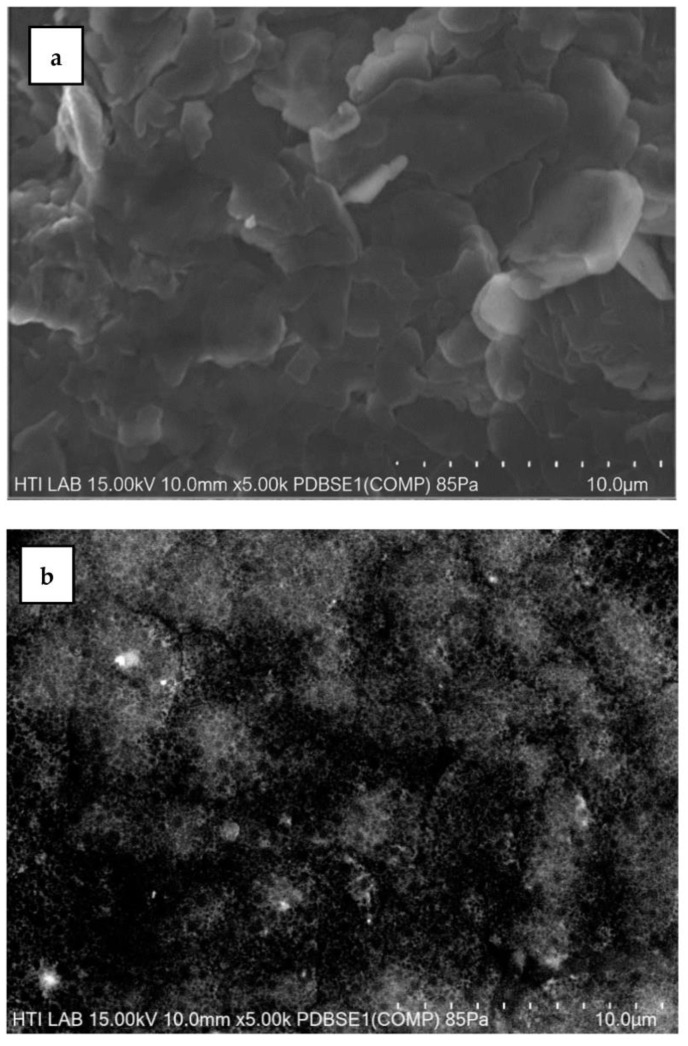
SEM images of PANI powders synthesized at (**a**) a stirring speed of 1200 rpm and (**b**) under optimized conditions.

The morphologies of the PANI powders is displayed in Figure 16. A PANI powder sample synthesized at a stirring speed of 1200 rpm was selected to represent micron-sized PANI and compared with a sample synthesized under optimized conditions. Figure 16a reveals that the former PANI has a flake shape with an average width of 3.282 nm and an average length of 8.358 nm. The morphology comprised the irregular shape of PANI, and the size is similar to the particle size identified using DLS. Similar outcomes were observed when PANI was synthesized without controlling the parameters, consequently producing a micron-sized PANI flake [18,45,54]. The mean diameter of PANI measured via DLS was 10 µm for the small population group, while the large population group had a diameter of 152 µm. Different morphologies of PANI are shown in Figure 16b, with a granular shape and good distribution. The granule has an average diameter of 695 nm, indicating that the nanosized PANI was synthesized under optimized conditions.

### 3.9. Effect of Particle Size on the Conductivity of PANI

**Figure 17:**
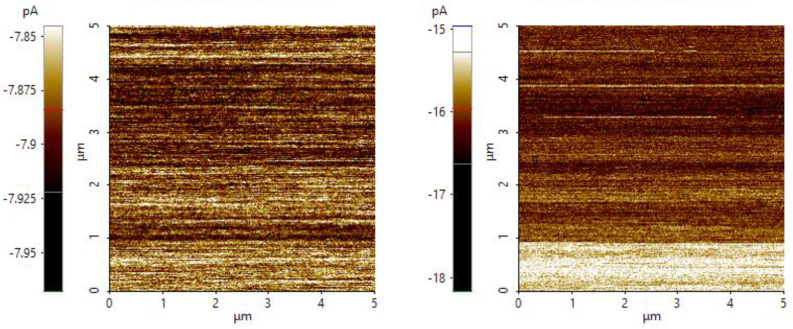
Images showing the current flow of a PANI powder synthesized at a stirring speed of 1200 rpm (**left**) and a PANI powder synthesized under optimized conditions (**right**).

**Figure 18:**
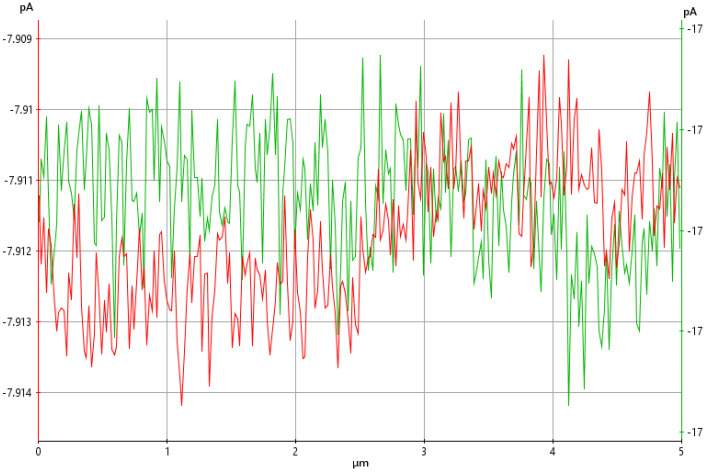
Low profiles of PANI powders synthesized at a stirring speed of 1200 rpm (red line) and under optimized conditions (green line) within a 5 µm^2^ scanned area.

AFM images representing the current flow profiles of PANI at different particle sizes are shown in Figure 17. The conductivity, indicated by the current flow range, is represented in the left panel. A lower current magnitude is indicated by white, and a higher magnitude is indicated by dark brown. The micron-sized PANI shows a lower range of current (−7.85 to −8.00 pA) flowing through the powder. The nanosized PANI shows a higher magnitude of current, with a range from −15 pA to −18 pA. The negative value of the current shows that PANI is connected to the back of a metallic disk [55]. The obvious difference in the magnitude of current flow for different particle sizes of PANI shows that particles with higher aspect ratios are important for yielding larger conductivity. Nanosized materials are favorable for many applications because their conductivity can be optimized. Current flow is improved by 108% when nanosized PANI is produced. The distribution of current flow is explained by the current flow profile across the 5 µm^2^ scanned area, as shown in Figure 18. The micron-sized PANI, represented by the red line, shows a current with an average value of −7.913 pA in the first half area and a decrease below −7.910 pA. The uniformity of current flow is more defined in the nanosized PANI.

## 4. Conclusions

For this paper, we studied the synthesis and characterization of nanosized PANI. The optimization of PANI synthesis parameters was performed to obtain PANI powders with sizes in the colloidal domain. The optimal parameters for synthesizing nanosized PANI are an initial molar ratio of 1, a stirring speed of 600 rpm, a temperature of 25 °C, and filtration and washing with the type of acid used to dope PANI, followed by acetone and distilled water. The use of Triton X-100 as the surfactant in PANI synthesis is preferable to SDS because of the smaller resulting particle size. The particle size of PANI corresponds to the dispersion stability in distilled water. The optimized parameters produced PANI with a size of 600 nm, which was well dispersed in neutral distilled water. The production of well-dispersed PANI is also apparent from our SEM analysis; meanwhile, an irregular shape was conferred to the PANI synthesized without controlling the parameters. The nanosized PANI had a higher conductivity than the micron-sized PANI. Producing nanosized PANI powder in chronological steps is important for increasing its conductivity. PANI produced in the nanoscale has potential uses in the fields of electronics and medicine.

## Figures and Tables

**Table 1:** List of chemicals.

Chemical	Usage	CAS No.	Brand
Aniline, >99.5%	Monomer	62-53-3	Sigma-Aldrich, St. Louis, MI, USA
Ammonium persulfate	Oxidizing agent	7787-54-0	R&M Chemicals, Calgary, AB, Canada
Hydrochloric acid, Fuming 37%	Doping agent	7647-01-0	R&M Chemicals
Sodium dodecyl sulfate, >99.5%	Anionic surfactant	151-21-3	Bendosen, Alen, Norway
Triton X-100, >99.5%	Nonionic surfactant	9036-19-5	Sigma-Aldrich
Acetone, >99.5%	Washing agent	67-64-1	Sigma-Aldrich
Methanol, >99.5%	Washing agent	67-56-1	Sigma-Aldrich
N-methylpyrrolidone, >99.5%	Solvent for analysis	872-50-4	Sigma-Aldrich

**Table 2:** Precursor method.

Chemicals	Chemicals
Initial molar ratio of aniline/APS (*r*)	1 (0.2 M:0.2 M)
Stirring speed during the synthesis period	800 rpm
Synthesis temperature	0–2 °C
Purification method	Filtration
Chemicals used for washing	Washing agent in the following order: 0.2 M HCl → 0.2 M acetone → Distilled water
Surfactant use	Not applied

**Table 3:** Yield of PANI synthesized with different *r* values.

Sample	Initial Molar Ratio (*r)*	Molar Concentration (M)		Weight of Aniline (g)	Weight of PANI (g)	% Yield
		Aniline	APS			
Equal molar	1	0.2	0.2	5.10	4.61	90.39
Unequal molar	3	0.3	0.1	6.12	2.12	34.72

**Table 4:** Particle size of PANI synthesized with different stirring speed values.

Sample	Population	Range (µm)	Median Diameter (µm)
PANI 600 rpm	1	2.41–29.91	10.10
	2	29.91–344.21	152.45
PANI 1200 rpm	1	4.47–44.94	15.17
	2	44.94–394.24	152.45

**Table 5:** Yield of PANI synthesized with different purification method.

Purification Method	Weight of Aniline (g)	Weight of PANI (g)	%Yield
Heating	10.50	10.06	95.99
Filtration		8.03	76.50

## Data Availability

The data presented in this study are available on request from the corresponding author.

## References

[1] Wang Y., Feng W. (2022). Conductive Polymers and Their Composites.

[2] Nezakati T., Seifalian A., Tan A., Seifalian A.M. (2018). Conductive Polymers: Opportunities and Challenges in Biomedical Applications. Chem. Rev..

[3] Nepomuceno N., Seixas A., Medeiros E., Mélo T. (2021). Evaluation of conductivity of nanostructured polyaniline/cellulose nanocrystals (PANI/CNC) obtained via in situ polymerization. J. Solid State Chem..

[4] Chauhan N.P.S., Mozafari M. (2019). Polyaniline: Future perspectives. Fundamentals and Emerging Applications of Polyaniline.

[5] Maruthapandi M., Saravanan A., Luong J.H.T., Gedanken A. (2020). Antimicrobial Properties of Polyaniline and Polypyrrole Decorated with Zinc-Doped Copper Oxide Microparticles. Polymers.

[6] Atta A., Abdelhamied M.M., Abdelreheem A.M., Berber M.R. (2021). Flexible Methyl Cellulose/Polyaniline/Silver Composite Films with Enhanced Linear and Nonlinear Optical Properties. Polymers.

[7] Zare E.N., Makvandi P., Ashtari B., Rossi F., Motahari A., Perale G. (2020). Progress in Conductive Polyaniline-Based Nanocomposites for Biomedical Applications: A Review. J. Med. Chem..

[8] Chauhan N.P.S., Mozafari M. (2019). Polyaniline: An introduction and overview. Fundamentals and Emerging Applications of Polyaniline.

[9] Miraftab R., Karimi B., Bahlakeh G., Ramezanzadeh B. (2017). Complementary experimental and quantum mechanics approaches for exploring the mechanical characteristics of epoxy composites loaded with graphene oxide-polyaniline nanofibers. J. Ind. Eng. Chem..

[10] Sunthar T.P.M., Marin E., Boschetto F., Zanocco M., Sunahara H., Ramful R., Kamei K., Zhu W., Pezzotti G. (2020). Antibacterial and Antifungal Properties of Composite Polyethylene Materials Reinforced with Neem and Turmeric. Antibiotics.

[11] Wu S.-Q., Wang J.-W., Wang G.-Q., Ren H. (2018). Enhanced dielectric properties of all-organic acrylic resin elastomer-based composite with doped polyaniline. Polym. Bull..

[12] Han J., Lu K., Yue Y., Mei C., Huang C., Wu Q., Xu X. (2019). Nanocellulose-templated assembly of polyaniline in natural rubber-based hybrid elastomers toward flexible electronic conductors. Ind. Crop. Prod..

[13] Zhang Y., Zhang J., Wang G., Zhang M., Luo Z. (2018). Preparation and Characterizing of PANI/PDMS Elastomer for Artificial Muscles. IOP Conf. Ser. Mater. Sci. Eng..

[14] Singh M., Sharib S.F.M., Mok K.L., Yatim A.H.M. (2019). Colloidal properties of precipitated calcium carbonate dispersion and its effect on prevulcanised natural rubber latex rheology and film tensile properties. J. Rubber Res..

[15] Saeb M.R., Zarrintaj P., Khandelwal P., Chauhan N.P.S. (2019). Synthetic route of polyaniline (I): Conventional oxidative polymerization. Fundamentals and Emerging Applications of Polyaniline.

[16] Zarrintaj P., Vahabi H., Saeb M.R., Mozafari M. (2019). Application of polyaniline and its derivatives. Fundamentals and Emerging Applications of Polyaniline.

[17] Namsheer K., Rout C.S. (2021). Conducting polymers: A comprehensive review on recent advances in synthesis, properties and applications. RSC Adv..

[18] Jamdegni M., Kaur A. (2020). Role of polarity of surfactants on the morphology of electrochemically synthesized polyaniline nanostructures: Towards faster and efficient electrochromic response. Thin Solid Films.

[19] Majeed A.H., Mohammed L.A., Hammoodi O.G., Sehgal S., Alheety M.A., Saxena K.K., Dadoosh S.A., Mohammed I.K., Jasim M.M., Salmaan N.U. (2022). A Review on Polyaniline: Synthesis, Properties, Nanocomposites, and Electrochemical Applications. Int. J. Polym. Sci..

[20] Ghorbankhani A., Zahedi A.R. (2022). Micro-cellular polymer foam supported polyaniline-nanofiber: Eco-friendly tool for petroleum oil spill cleanup. J. Clean. Prod..

[21] Bhandari S. (2018). Polyaniline: Structure and properties relationship. Polyaniline Blends, Composites, and Nanocomposites.

[22] Jarach N., Meridor D., Buzhor M., Raichman D., Dodiuk H., Kenig S., Amir E. (2020). Hybrid Antibacterial and Electro-conductive Coating for Textiles Based on Cationic Conjugated Polymer. Polymers.

[23] Zhang N., Cao H. (2020). Enhancement of the Antibacterial Activity of Natural Rubber Latex Foam by Blending It with Chitin. Materials.

[24] Al-Hada N.M., Al-Ghaili A.M., Baqer A.A., Saleh M.A., Kasim H., Saion E., Liu J., Jihua W. (2020). Radiation-induced synthesis, electrical and optical characterization of conducting polyaniline of PANI/ PVA composites. Mater. Sci. Eng. B.

[25] German N., Popov A., Ramanaviciene A., Ramanavicius A. (2017). Evaluation of enzymatic formation of polyaniline nanoparticles. Polymer.

[26] Wang C., Guo Z., Hong R., Gao J., Guo Y., Gu C. (2018). A novel method for synthesis of polyaniline and its application for catalytic degradation of atrazine in a Fenton-like system. Chemosphere.

[27] Fang F.F., Dong Y.-Z., Choi H.J. (2018). Effect of oxidants on morphology of interfacial polymerized polyaniline nanofibers and their electrorheological response. Polymer.

[28] Rashid I.A., Irfan M.S., Gill Y.Q., Nazar R., Saeed F., Afzal A., Ehsan H., Qaiser A.A., Shakoor A. (2020). Stretchable strain sensors based on polyaniline/thermoplastic polyurethane blends. Polym. Bull..

[29] Srinivas C.H., Srinivasu D., Kavitha B., Narsimlu N., Kumar K.S. (2012). Synthesis and characterization of nano size conducting polyaniline. IOSR J. Appl. Phys..

[30] Prasutiyo Y.J., Manaf A., Hafizah M.A.E. (2020). Synthesis of polyaniline by chemical oxidative polymerization and characteristic of conductivity and reflection for various strong acid dopants. J. Phys. Conf. Ser..

[31] Aribowo S., Hafizah M.A.E., Manaf A., Andreas A. (2018). Study of aniline polymerization reactions through the particle size formation in acidic and neutral medium. AIP Conference Proceedings.

[32] Hafizah M.A.E., Riyadi A.F., Manaf A., Andreas A. (2019). Particle size reduction of polyaniline assisted by anionic emulsifier of sodium dodecyl sulphate (SDS) through emulsion polymerization. IOP Conference Series: Materials Science and Engineering.

[33] Lorenzen A.L., Rossi T.S., Riegel-Vidotti I.C., Vidotti M. (2018). Influence of cationic and anionic micelles in the (sono)chemical synthesis of stable Ni(OH)_2_ nanoparticles: “In situ” zeta-potential measurements and electrochemical properties. Appl. Surf. Sci..

[34] Banjar M.F., Suphi H.D., Sarizan M.I., Yahaya A.N.A., Khalil N.A., Singh M., Zulkifli M. (2021). Fundamental study of colloidal stability and dispersion of novel nanosized conductive polyaniline (PANI)/prevulcanised latex (PVL) film for antimicrobial applications. IOP Conf. Ser. Mater. Sci. Eng..

[35] Stejskal J., Gilbert R.G. (2002). Polyaniline. Preparation of a conducting polymer (IUPAC Technical Report). Pure Appl. Chem..

[36] Mahitha P., Sneha K., Advaith P., Sultan K.R., Sajith M., Jose B., Hema S., Sambhudevan S., Shankar B. (2019). Conducting polyaniline based rubber nanocomposites—Synthesis and characterization studies. Mater. Today Proc..

[37] Zarrintaj P., Khalili R., Vahabi H., Saeb M.R., Ganjali M.R., Mozafari M. (2019). Polyaniline/metal oxides nanocomposites. Fundamentals and Emerging Applications of Polyaniline.

[38] (2016). Standard Practices for General Techniques of Infrared Quantitative Analysis.

[39] Reddy K.R., Hemavathi B., Balakrishna G.R., Raghu A.V., Naveen S., Shankar M.V. (2018). Organic conjugated polymer-based functional nanohybrids: Synthesis methods, mechanisms and its applications in electrochemical energy storage supercapacitors and solar cells. Polymer Composites with Functionalized Nanoparticles: Synthesis, Properties, and Applications.

[40] Barbero C.A., Acevedo D.F. (2022). Mechanochemical Synthesis of Polyanilines and Their Nanocomposites: A Critical Review. Polymers.

[41] Batista A.F., Rodrigues-Siqueli A.C., de Oliveira A.P.S., Petraconi G., Baldan M.R. (2022). Facile synthesis of polyaniline catalyzed by carbon fiber for supercapacitor applications. Synth. Met..

[42] Mahmoud M.E., Saad E.A., El-Khatib A.M., Soliman M.A., Allam E.A., Fekry N.A. (2018). Green solid synthesis of polyaniline-silver oxide nanocomposite for the adsorptive removal of ionic divalent species of Zn/Co and their radioactive isotopes 65Zn/ 60Co. Environ. Sci. Pollut. Res..

[43] Zhu H., Peng S., Jiang W. (2013). Electrochemical Properties of PANI as Single Electrode of Electrochemical Capacitors in Acid Electrolytes. Sci. World J..

[44] Kamarudin S., Rani M.S.A., Mohammad M., Mohammed N.H., Su’Ait M.S., Ibrahim M.A., Asim N., Razali H. (2021). Investigation on size and conductivity of polyaniline nanofiber synthesised by surfactant-free polymerization. J. Mater. Res. Technol..

[45] Stejskal J., Sapurina I., Trchová M. (2010). Polyaniline nanostructures and the role of aniline oligomers in their formation. Prog. Polym. Sci..

[46] Sajith M., Jose B., Sambhudevan S., Sreekala C.O., Shankar B. (2019). Effect of matrix type and doping on polyaniline based natural rubber nanocomposites. AIP Conference Proceedings.

[47] Lin C.-W., Mak W.H., Chen D., Wang H., Aguilar S., Kaner R.B. (2019). Catalytic Effects of Aniline Polymerization Assisted by Oligomers. ACS Catal..

[48] Yang Y.-J., Corti D.S., Franses E.I. (2017). Effect of Triton X-100 on the stability of titania nanoparticles against agglomeration and sedimentation: A masked depletion interaction. Colloids Surf. A Physicochem. Eng. Asp..

[49] Shah R., Eldridge D., Palombo E., Harding I. (2014). Optimisation and Stability Assessment of Solid Lipid Nanoparticles Using Particle Size and Zeta Potential. J. Phys. Sci..

[50] Bednarczyk K., Matysiak W., Tański T., Janeczek H., Schab-Balcerzak E., Libera M. (2021). Effect of polyaniline content and protonating dopants on electroconductive composites. Sci. Rep..

[51] Neelgund G.M., Oki A. (2011). A facile method for the synthesis of polyaniline nanospheres and the effect of doping on their electrical conductivity. Polym. Int..

[52] Yang J., Ding Y., Chen G., Li C. (2007). Synthesis of conducting polyaniline using novel anionic Gemini surfactant as micellar stabilizer. Eur. Polym. J..

[53] Swaruparani H., Basavaraja S., Basavaraja C., Huh D.S., Venkataraman A. (2010). A new approach to soluble polyaniline and its copolymers with toluidines. J. Appl. Polym. Sci..

[54] Sapurina I., Stejskal J. (2008). The mechanism of the oxidative polymerization of aniline and the formation of supramolecular polyaniline structures. Polym. Int..

[55] Mikulik D., Ricci M., Tutuncuoglu G., Matteini F., Vukajlovic J., Vulic N., Alarcon-Llado E., i Morral A.F. (2017). Conductive-probe atomic force microscopy as a characterization tool for nanowire-based solar cells. Nano Energy.

